# Farnesol-Induced Apoptosis in *Candida albicans* Is Mediated by Cdr1-p Extrusion and Depletion of Intracellular Glutathione

**DOI:** 10.1371/journal.pone.0028830

**Published:** 2011-12-19

**Authors:** Jingsong Zhu, Bastiaan P. Krom, Dominique Sanglard, Chaidan Intapa, Clinton C. Dawson, Brian M. Peters, Mark E. Shirtliff, Mary Ann Jabra-Rizk

**Affiliations:** 1 Department of Oncology and Diagnostic Sciences, University of Maryland, Baltimore, Maryland, United States of America; 2 Department of Preventive Dentistry, Academic Centre for Dentistry Amsterdam (ACTA), University of Amsterdam and Free University Amsterdam, Amsterdam, The Netherlands; 3 Institute of Microbiology, University of Lausanne and University Hospital Center, Lausanne, Switzerland; 4 Department of Oral Diagnostic Science, Faculty of Dentisty, Naresuan University, Phitsanulok, Thailand; 5 Graduate Program in Life Sciences, Microbiology and Immunology Program, School of Medicine, University of Maryland, Baltimore, Maryland, United States of America; 6 Department of Microbial Pathogenesis, Dental School, University of Maryland, Baltimore, Maryland, United States of America; 7 Department of Microbiology and Immunology, School of Medicine, University of Maryland, Baltimore, Maryland, United States of America; University of Minnesota, United States of America

## Abstract

Farnesol is a key derivative in the sterol biosynthesis pathway in eukaryotic cells previously identified as a quorum sensing molecule in the human fungal pathogen *Candida albicans*. Recently, we demonstrated that above threshold concentrations, farnesol is capable of triggering apoptosis in *C. albicans*. However, the exact mechanism of farnesol cytotoxicity is not fully elucidated. Lipophilic compounds such as farnesol are known to conjugate with glutathione, an antioxidant crucial for cellular detoxification against damaging compounds. Glutathione conjugates act as substrates for ATP-dependent ABC transporters and are extruded from the cell. To that end, this current study was undertaken to validate the hypothesis that farnesol conjugation with intracellular glutathione coupled with Cdr1p-mediated extrusion of glutathione conjugates, results in total glutathione depletion, oxidative stress and ultimately fungal cell death. The combined findings demonstrated a significant decrease in intracellular glutathione levels concomitant with up-regulation of *CDR1* and decreased cell viability. However, addition of exogenous reduced glutathione maintained intracellular glutathione levels and enhanced viability. In contrast, farnesol toxicity was decreased in a mutant lacking *CDR1*, whereas it was increased in a *CDR1*-overexpressing strain. Further, gene expression studies demonstrated significant up-regulation of the *SOD* genes, primary enzymes responsible for defense against oxidative stress, with no changes in expression in *CDR1*. This is the first study describing the involvement of Cdr1p-mediated glutathione efflux as a mechanism preceding the farnesol-induced apoptotic process in *C. albicans*. Understanding of the mechanisms underlying farnesol-cytotoxicity in *C. albicans* may lead to the development of this redox-cycling agent as an alternative antifungal agent.

## Introduction

*Candida albicans* is the most important human fungal pathogen causing diseases varying from superficial mucosal infections to life-threatening systemic disorders [1], [2]. As a polymorphic species, *C. albicans* is capable of morphological switching, a transition central to its pathogenesis and ability to form biofilms on a variety of surfaces, including host tissue [3]–[5]. Farnesol, synthesized from farnesyl pyrophosphate, an intermediate in the sterol biosynthetic pathway, was identified as a quorum sensing molecule secreted by *C. albicans*, able to prevent yeast-to-hyphal conversion and biofilm formation [6]–[9]. Recently, through assessment of classical apoptotic markers and global proteomic analysis we demonstrated that above threshold concentrations, farnesol induces apoptosis in *C. albicans* and in human oral tumor cells [10], [11]. Among the apoptotic markers identified were the activation of intracellular caspases, a class of proteolytic enzymes which when activated, convey an apoptotic signal and induce apoptosis [10]–[12]. However, to date, the underlying mechanism of farnesol cytotoxicity in eukaryotic cells is yet to be fully elucidated.

Apoptosis is a naturally occurring developmental process triggered by various extracellular and intracellular stimuli. This event causes mitochondrial membrane permeability and dissipation of the electrochemical gradient, uncoupling of the respiratory chain and hyperproduction of superoxide anions culminating in cell death [13], [14]. Oxidative stress is well recognized as a universal trigger for apoptosis [15]. However, apoptosis has also been reported to be associated with glutathione depletion which may result in oxidative stress by altering the cell reducing power with extrusion of glutathione out of the cell [15], [16]. Glutathione, present mainly in its reduced form (GSH) is a ubiquitous thiol-containing tripeptide composed of cysteine, glutamic acid and glycine, synthesized in the cell by the sequential actions of a series of six-enzyme-catalyzed reactions termed the γ-glutamyl cycle [15]–[19]. Glutathione plays a key role in cellular resistance against oxidative damage through the detoxification of free radicals and naturally occurring deleterious compounds, as well as a variety of xenobiotics [16]–[18], [20]. Therefore, the response of a cell to a stress involves changes in glutathione content, which is consumed in reactions that protect the cell by removing damaging compounds [16].

Glutathione detoxifies xenobiotics and toxic compounds through irreversible conjugation, concomitant with its conversion to the oxidized form, a reaction catalyzed by glutathione peroxidase (GPX). This reaction results in the formation of glutathione S-conjugates which are ultimately excreted from the cell [16], [17], [19]. The oxidized form, glutathione disulfide (GSSG), normally represents <2% of the total glutathione pool with the reduced GSH predominating over GSSG [17], [18]. Perturbations of the GSH/GSSG ratio can affect the redox status and induce cellular apoptosis and thus, the ratio is generally regulated tightly to maintain cellular homeostasis [17], [18]. Therefore, the intracellular content of GSH is a function of the balance between depletion and synthesis [17], [18]. Cells use two competing mechanisms to maintain low levels of GSSG and high intracellular ratio of GSH to GSSG. The first mechanism is a recycling reaction where GSSG is reverted to GSH by the action of the enzyme glutathione reductase (GLR) which rapidly converts GSSG to GSH. The second mechanism involves cellular export of S-conjugates by membrane efflux pumps [16], [17], [20]. In *C. albicans*, the importance of glutathione function to the growth of the fungus was demonstrated by the studies of Baek *et al.* [19], where the generation of a null mutant strain deficient in glutathione synthesis led to an increased generation of ROS, as well as entry into apoptosis which could be rescued by supplementing with GSH.

In mammalian cells, the release of glutathione S-conjugates from cells is an ATP-dependent process mediated by integral plasma membrane glycoproteins (MRP) belonging to the ubiquitous ATP-binding cassette superfamily of transporters (ABCT) [21], [22]. These transporters have been shown to export a wide range of substrate molecules, including lipids and hydrophobic compounds [22], [23]. Many lipophilic compounds conjugated with glutathione are substrates for the MRP family and the over-expression of *MRP1* was shown to increase ATP-dependent glutathione S-conjugate carrier activity, whereas *MRP1* inhibitors diminish cellular extrusion of GSSG [21], [24]. Therefore, these efflux pumps play a decisive role in detoxification and defense against oxidative stress. In *C. albicans*, the ABC transporter genes *CDR1* and *CDR2* (Candida Drug Resistance) encode ATP-dependent plasma membrane efflux pumps [25], [26]. The overexpression of these genes is considered to be a major component of antifungal drug resistance and their deletion has been shown to result in hypersensitivity [25]. These proteins (Cdr1p and Cdr2p) are homologues of the mammalian *MRP1*, sharing several characteristics with respect to substrate binding and counteracting inhibitory effects of endogenous metabolites [25], [26]. Therefore, due to their involvement in cellular detoxification, stress response and drug resistance, members of this ubiquitous superfamily of ABCT act at crossroads of vital cellular processes, constituting a first line of defense against environmental toxins [27].

As a lipophilic hydrophobic cation and a precursor in the sterol pathway, farnesol would classify as a compound that conjugates with GSH, particularly given its known deleterious effect on cells [10], [11]. Therefore, conjugation of farnesol with intracellular GSH thus disrupting the intracellular redox equilibrium would provide a pathway ultimately leading to cellular apoptosis. However, this potential mechanism for farnesol cytotoxicity has not been previously investigated. To that end, in this current study, we aimed to expand on our initial work demonstrating a farnesol-induced apoptotic process in *C. albicans*. Specifically, experiments were designed to validate the hypothesis that farnesol exerts its cytotoxic effect by altering, either directly or indirectly, the cellular redox status through consumption of reduced GSH, modulation of *CDR1* induction and disruption of the intracellular redox homeostatis predisposing the cell to apoptosis.

## Materials and Methods

### Strains, growth conditions and reagents

Table 1 describes the *C. albicans* strains used in this study. Strains DSY448 (lacking *CDR1*), DSY653 (lacking *CDR2*) and DSY653 (lacking *CDR1* and *CDR2)* were constructed as previously described by Sanglard *et al.* [28], [29]. The Cdr1p-overexpressing strain FL3 expressing *CDR1* under the control of the *HEX1* promoter and its control parent strain DL1 lacking the *CDR1* construct were constructed as previously described by Niimi *et al*. [30]. The wild-type strain SC5314 and CAF2-1 were used as parent strains (CA). The *CDR1* complemented strain was constructed for this study as described below. Strains were grown in YPD broth (Difco Laboratories, Detroit, MI) overnight at 30°C with shaking and cells were equilibrated in fresh media to an optical density of 1.0 at OD_600_. Cells were grown in YPD in the presence of farnesol for 3 h or 18 h exposure. Cells were used at final cell density of 2×10^7^ cells/ml in PBS unless otherwise stated.

**Table 1:** Isogenic *C. albicans* strains used in this study.

Strain	Genotype	Parent	Reference
SC5314	Wild-typ2		Gillum *et al*. 1984
CAF2-1	*ura3Δ*::*imm434/URA3*	SC5314	Fonzi *et al.* 1993
CAI4	*ura3Δ*::*imm434/ura3Δ*::*imm434*	CAF2-1	Fonzi *et al.* 1993
DSY448	*cdr1Δ*::*hisG-URA3-hisG/cdr1Δ*::*hisG*	CAI4	Sanglard *et al.* 1996
DSY449	*cdr1Δ*::*hisG/cdr1Δ*::*hisG*	DSY448	Sanglard *et al.* 1996
DSY653	*cdr2Δ*::*hisG-URA3-hisG/cdr2Δ*::*hisG*	CAI4	Sanglard *et al.* 1997
DSY4310	*ura3Δ*::*imm434*/*ura3Δ*::*imm434, RP10*::Clp10	CAI4	this work
DSY4480	*cdr1Δ*::*hisG/cdr1Δ*::*hisG, RP10*::Clp10	DSY449	this work
DSY4881	*cdr1Δ*::*hisG/cdr1Δ*::*hisG*::*CDR1*	DSY449	this work
FL3	*cdr1Δ*::*hisG/cdr1Δ*::*hisG,* pMN9131	CAI4	Niimi *et al.* 2004
DL1	*cdr1Δ*::*hisG/cdr1Δ*::*hisG,* pRC2312	CAI4	Niimi *et al.* 2004

Farnesol, reduced glutathione, 3-(4,5-dimethylthiazol-2-yl)-2,5-diphenyltetrazolium bromide, N-acetylglucosamine (GlcNAc), Total Glutathione, Glutathione Peroxidase and Glutathione Reductase kits were obtained from Sigma (Sigma-Aldrich Chemical, St. Louis, MO). Farnesol was obtained as a 3 M stock solution and diluted to a 30 mM solution in 100% methanol. Methanol was incorporated in the control experiments however previous experiments had shown that methanol did not have an effect on cell viability at the concentration used in these experiments [6], [11]. To evaluate whether GSH supplementation salvages cells from effects of farnesol, 25 mM GSH (in dH_2_O) was incorporated in experiments where indicated. Reduced glutathione had no effect on survival of control cells. All experiments were performed on three separate occasions.

### Revertant construction and confirmation of complementation

The *CDR1* gene including 815-bp upstream and 295-bp downstream flanking regions was amplified from *C. albicans* SC5314 genomic DNA with primers CDR1-SalI (5′-TTTTGTCGACAATTTCAACAATTAACTTCA-3′) and CDR1-ApaI (5′-CTTGGGCCCAATAATTCAATAATACACAATTT-3′). The resulting fragment was cloned into ClP10 [31] digested by SalI and ApaI to yield pDS1765. This plasmid was linearized with NsiI, which cuts within the *CDR1* promoter to facilitate integration in the *CDR1* genomic locus and next transformed by a LiAc method into the *C. albicans* strain DSY449, which is the *ura3* derivative from DSY448 [28]. CAF4-2 and DSY449 were transformed with CIP10 digested with StuI for integration at the neutral *RP10* locus to yield DSY4310 and DSY4480, respectively.

Complementation of *CDR1* inactivation was analyzed by drug resistance phenotype. Briefly, following selection of transformants in YNB selective medium lacking uracil, *C. albicans* strains were serially diluted in PBS and 5 µl of each dilution were deposited onto agar plates containing 100 µg/ml fluphenazine and plates were incubated at 35°C for 48 h. In addition, one of the positive transformants (DSY4481) was further tested by Western analysis following growth for 30 min in the absence or presence of fluphenazine (20 µg/ml). Proteins were extracted from cells and immunoblotting performed using an antibody against Cdr1p as previously described [32]. As observed in Fig.S1A, wild type fluphenazine susceptibility was restored in the revertant (DSY4481). Similarly, Western analysis showed a signal corresponding to Cdr1p in DSY4481 as well as the wild type control (DSY4310) which was absent in the *CDR1* mutant strain (DSY4480) (Fig.S1B).

### Total cellular glutathione content

The total cellular glutathione content was measured in farnesol-exposed cells (2×10^8^ cells) in the presence and absence of exogenous glutathione using Glutathione Assay Kit (Sigma-Aldrich) according to manufacturer instructions. Briefly, cells were harvested, washed with PBS and 3 volumes of 5% SSA solution was added to the cell pellet. The suspension was frozen in liquid nitrogen and thawed in 37°C water bath and suspension centrifuged at 10,000×g for 10 min. 10 µl of supernatant was used to mix with 150 µl working mixture for 5 minutes before 50 µl of NADPH was added. Absorbance at 412 nm was monitored at 1 minute intervals for a total of 5 min. The content of total glutathione was quantified by comparison with known glutathione standards. Results were expressed as percent decrease in intracellular glutathione levels in farnesol-exposed cells compared to control cells.

### MTS viability assay

Cell proliferation of farnesol-treated cells was assessed using the MTS Tetrazolium-Based Proliferation Assay (Promega, Madison, WI) according to manufacturer directions. Farnesol was added to the cells at final concentrations of 10, 40, 100, 200 and 300 µM in the wells of a 96-well microtiter plate in PBS. Control reactions with cells and no farnesol were included. Plates were incubated for 3 h or 18 h at 30°C with shaking. Following incubation, 20 µl of the MTS reagent was added to each well and plates were incubated at 30°C for 4 h or until color fully developed. Following color development, each sample was transferred to a fresh plate and colorimetric change at 490 nm (A_490_) was measured with a microtiter plate reader (Titertrek, Multiskan MCC1340). MTS assay was also performed with glutathione supplementation. In addition, *C. albicans* cell density and time course experiments were also performed using the MTS assay where viability of *C. albicans* was assessed at different cell densities (1×10^6^–2×10^8^ cells/ml) and exposure times.

### Viability assay by plate dilution

In addition to the MTS assay, viability of *C. albicans* following increasing concentrations of farnesol was also assessed based on colony forming units (CFU) counts. In these experiments cells were incubated with farnesol as described above. Following incubation 360 µl of YPD broth was added to each well and 10 µl of cell suspension was spread on YPD agar plates. Following 24 h incubation, colonies were counted and farnesol killing was expressed as percent killing based on decrease in CFU counts compared to CFUs of cells not exposed to farnesol. Viability assays were also performed with glutathione supplementation.

### Overexpression of *CDR1* in *C. albicans*

Overexpression of *CDR1* was induced in *C. albicans* FL3 strain as previously described [30]. Briefly, cells were grown in CSM-URA (Bio 101, Vista, CA) medium (containing 1% glucose) at 30°C for 16 h, subcultured into the same medium at an initial density of 2×10^7^ cells/ml and incubated at 30°C until cells reached a density of 8×10^7^ cells/ml. Cells were then washed three times in sterile water and resuspended at cell density of 6×10^7^cells/ml and starved of glucose by incubation at 30°C for 16 h. The cells were resuspended at cell density of 4×10^7^ cells/ml in CSM-URA medium containing GlcNAc (25 mM) as carbon source and incubated at 27°C for 1.5 h to induce the *HEX1* promoter and *CDR1* expression (a temperature of 27°C was used to ensure that all cells were in the yeast morphology; expression of *CDR1* mRNA was previously shown to be induced rapidly, within 0.5 h) [30]. Following incubation, cells were harvested, washed and tested with farnesol in the MTS assay as described above. The DL1 control parent strain with matched auxotrophies but lacking the *CDR1* construct was grown under identical conditions. To check whether *CDR1* overexpression conferred resistance to fluconazole, fluconazole susceptibility testing was performed and interpreted according to the guidelines outlined in The National Committee for Clinical Laboratory Standards broth microdilution protocol (NCCLS) [33]. The results demonstrated that although both MIC values were within the susceptible range, the MIC after induction was consistently higher (0.5 µg/ml) than that for the strain prior to induction (0.25 µg/ml) or to CA on all occasions tested.

To confirm the overexpression of *CDR1*, gene expression studies were performed on RNA extracted from FL3 and DL1 strains following the induction procedure. Analysis was also performed on CA and *cdr1* strains. RT-PCR was performed as described below in Gene Expression section.

### GSH/GSSG homeostatis and redox balance

Farnesol-induced disruption in intracellular redox balance was assessed using the GSH/GSSG, performed as previously described by Gonzalez-Parraga *et al.* [34] with some modifications. Briefly, cells were exposed to farnesol in PBS for 3 h and 18 h. Farnesol-exposed cells were harvested at the indicated times, washed and resuspended in 2 ml of cold 5% meta-phosphoric acid. The cellular suspensions were transferred into small pre-cooled tubes with glass beads in a bead beater and were subjected to 6 cycles of 45 seconds each. Following centrifugation at 10000 g for 5 min, 500 µl of supernatant was mixed with 750 µl of 0.5 M K-phosphate buffer (pH 7.5) and 25 µl of dH_2_O. This sample was used for total glutathione assay. Another 500 µl of supernatant was mixed with 750 µl of 0.5 M K-phosphate buffer (pH7.5) and 25 µl of 2-vivylpyridine until an emulsion was formed and samples were incubated for 1 h at room temperature. Following incubation, 150 µl mixture of 0.2 mM NADPH, 50 M K-phosphate buffer (pH7.5), 5 mM EDTA, 0.6 mM DTNB and 3 units of glutathione reductase enzyme was mixed with 5 µl of extracted samples in 96-well microtiter plates. The reaction rate was monitored by measuring the change in absorbance at 412 nm for 1 min and a standard curve was developed based on glutathione in the range of 0–100 µmol/ml.

### Confocal scanning laser microscopy

Experiments were performed on cells exposed to 40 and 200 µM farnesol for 3 h and 18 h. For these experiments, cells were grown in YPD at final cell density of 2×10^7^ cells/ml in the presence and absence of farnesol or exogenous reduced GSH. Following incubation at 30°C with shaking, cells were harvested, washed in PBS and processed for ROS accumulation and caspase activation and observed with a Zeiss Axiovert 100 confocal microscope (with video capture system, automatic camera and image analysis hardware and software) using 20×, 40× and 100× oil immersion objectives. Imaging of stained cells was accomplished by using a Cy2/Cy3 multitrack filter set. Images were processed for display by using Axiovision 3.× software (Zeiss). Quantification was performed by scoring green fluorescent cells (ROS accumulation, caspase activation) and red fluorescent cells (necrotic cells) relative to all cells in 10 fields.

### ROS accumulation

Accumulation of intracellular reactive oxygen species (ROS) was assayed using a mixture of 100 µl of the fluorescent probe dichlorodihydrofluorescein diacetate (5 mM in EtOH) (DCDHF-DA; Molecular Probes), 100 µl of the chitin-binding stain calcofluor white (1 mg/ml) (Sigma-Aldrich) which allows visualization of all healthy cells and propidium iodide (Sigma-Aldrich) which stains dead cells. The mixture (4.5 µl) was added to cells and incubated for 30 min in the dark. Following incubation, cells were collected by centrifugation (5 min, 4000× g), washed with PBS and re-suspended in 50 µl PBS.

### Detection of caspase activation

Activated caspases in the farnesol-exposed cells with and without glutathione supplementation were detected microscopically using FLICA Apoptosis Detection Kits (Immunochemistry Technologies, LLC, Bloomington, MN) according to manufacturer directions. Cells with intracellular active caspases fluoresce green whereas non-apoptotic cells appear unstained.

### Glutathione peroxidase (GPX) activity

Glutathione peroxidase activity in 3 h or 18 h farnesol-exposed cells in the presence and absence of exogenous glutathione was measured using the colorimetric Glutathione Peroxidase Cellular Activity Assay Kit (Sigma) according to manufacturer directions. The reaction is based on the reduction of hydrogen peroxide and organic peroxides to the corresponding stable alcohols and water by cellular glutathione in the presence of glutathione peroxidase. Cells (2×10^8^) were resuspended in 100 µl of assay buffer and cell suspension was frozen in liquid nitrogen and thawed at 37°C twice then centrifuged at 10000×g for 10 min. Supernatant was recovered and 50 µl of the sample was mixed with 0.25 mM NADPH and 0.21 mM reduced glutathione and 0.5 units/ml glutathione reductase in buffer. The reaction was started by 300 µM t-Bu-OOH. Absorbance at 340 nm was recorded at 10 seconds interval for 6 times after 15 seconds initial delay. The activity of glutathione peroxidase in the sample expressed in mmol/min/ml was calculated using the formula: (Δ_blank_-Δ_sample_)/(ε^mM^×0.05), where ε^mM^ = 6.22.

### Glutathione reductase (GLR) activity

Glutathione reductase activity in 3 h or 18 h farnesol-exposed cells in the presence and absence of exogenous glutathione was measured using the colorimetric Glutathione Reductase Assay Kit (Sigma) according to manufacturer directions based on the reduction of GSSG to GSH by NADPH in the presence of glutathione reductase with controls included. 2×10^8^ cells were re-suspended in 100 µl of assay buffer. The cell suspension was frozen in liquid nitrogen and thawed at 37°C twice then centrifuged at 10000×g for 10 min. Supernatant was recovered and 50 µl of the sample was mixed with 2 mM Oxidized Glutathione and 3 mM DTNB in buffer. The reaction was started by the addition of 2 mM NADPH. Absorbance at 412 nm was reported at 10 seconds intervals for a total of 11 readings. The concentration of the enzyme reported as mmol/min/ml/10^8^ cells was calculated using the formula above.

### Gene expression analysis

For detection of gene transcripts, gene-specific primer pairs (Table 2) were designed based on gene sequences in GenBank. Total RNA was isolated from cultures using an Invisorb® Spin Cell Total RNA mini kit (Invitek, Berlin, Germany) after grinding cells in liquid nitrogen. DNA was removed by DNase (New England Biolabs) treatment followed by clean up using the RNeasy protocol (Qiagen Benelux BV, Venlo, The Netherlands). The RNA concentration was determined using a Nanodrop UV/VIS spectrophotometer (Isogen-Biosolutions Inc., Maarsen, The Netherlands). Absence of genomic DNA was confirmed by performing PCR using an *ACT1* primer pair (Table 2) prior to the reverse transcription step. Reverse transcription was performed on 1 µg of total RNA using an iScript™ cDNA synthesis kit (Biorad, Veenendaal, the Netherlands) as specified. Synthesized cDNA was diluted 1∶20 in DEPC treated water and stored at −80°C until needed. Sample preparation was performed using a CAS-1200™ pipetting robot (Corbett Life Science, Sydney, Australia). Real time PCR was performed on a MyCycler real time thermocycler (Biorad, Veenendaal, the Netherlands) using ABsolute QPCR SYBR Green mix (Abgene, Epsom, United Kingdom) and primer pairs listed in Table 2. Amplification was achieved using the following cycle settings: 15 min 95°C followed by 35 cycles of 95°C 1 min, 58°C 30 s, 72°C 30 s. After the amplification a melt curve was analyzed to ensure the absence of primer dimers. Expression was calculated using the *ACT1* as reference gene.

**Table 2:** Primer sequences used in RT-PCR.

*Primer name*	*Sequence (5′-to-3′)*
EFB1-F	GAACGAATTCTTGGCTGAC
EFB1-B	CATCAGAACCGAACAAGTC
CDR1-F	GAATCGGGATTCAATTGGTC
CDR1-B	GAATCGGGATTCAATTGGTC
SOD1-F	TTGAACAAGAATCCGAATCC
SOD1-B	AGCCAATGACACCACAAGCAG
SOD2-F	ACCACCCGTGCTACTTTGAAC
SOD2-B	GCCCATCCAGAACCTTGAAT
SOD3-F	ACAATGCCGCTATTGACGCA
SOD3-B	AGCCCAGTTGATCACGTTCCA
SOD4-F	CCAGTGAATCATTTGAAGTTG
SOD4-B	AGAAGCACTAGTTGATGAACC
GLR1-F	AATTGGTGTTTTCCGCTGAC
GLR1-B	AACCCCAATGTAACCAGCAC
GPX2-F	TGTGTGGGTTCACACCTCAA
GPX2-B	ATGGGGAAACTGACACCAAA

In addition to qRT-PCR, gene expression analysis was also performed using cDNA synthesis/RT-PCR. Reactions were analyzed on a 1% agarose gels containing 0.5grams/ml ethidium bromide and bands were visualized with a MultiImager scanner (Bio-Rad Laboratories, Hercules California) and images analyzed with the PDQuest version 7.0 (BioRAd).

### Statistical analysis

All experiments were performed in triplicate on 3 different occasions. Student's t-Test was employed to assess the statistical significance of treated versus untreated samples along with standard error. A statistically significant difference was considered to be present at p<0.05.

## Results

### Effect of farnesol on *CDR1* gene expression

**Figure 1:**
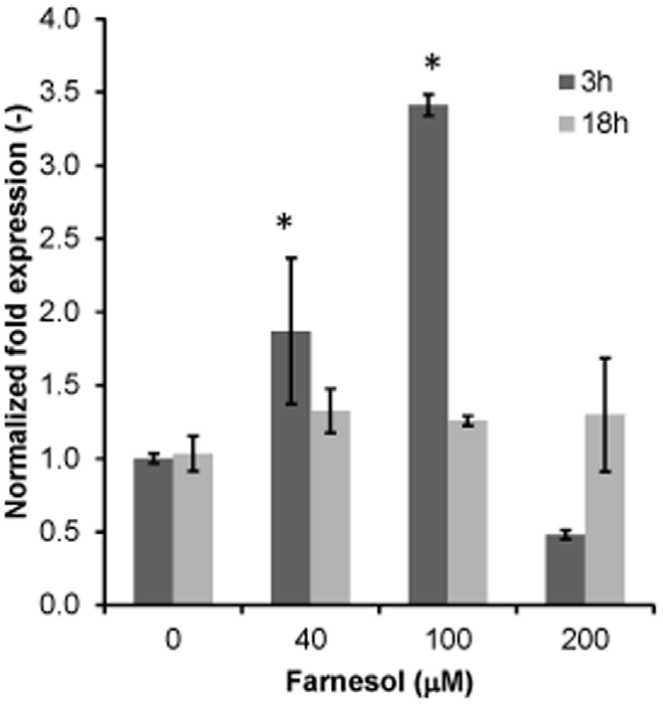
Effect of farnesol on the expression of *CDR1* in CA following 3 and 18 h exposure to increasing concentrations of farnesol. Farnesol-concentration and exposure time-dependent up-regulation of *CDR1*. Expression is normalized against *ACT1* expression. Data represent the average of 3 independent experiments performed in triplicate. Error bars indicate the standard errors of the means. p<0.05.

To determine whether farnesol exposure modulates the expression of the efflux pump *CDR1*, gene expression studies were performed. Results from these experiments demonstrated that farnesol exposure resulted in significant increase in the expression of *CDR1* relative to the untreated control cells (Fig. 1). This increase in expression was not changed by the addition of GSH (data not shown). The profile of gene expression was similar to that obtained by RT-PCR analysis (data not shown).

### Total cellular glutathione content

**Figure 2:**
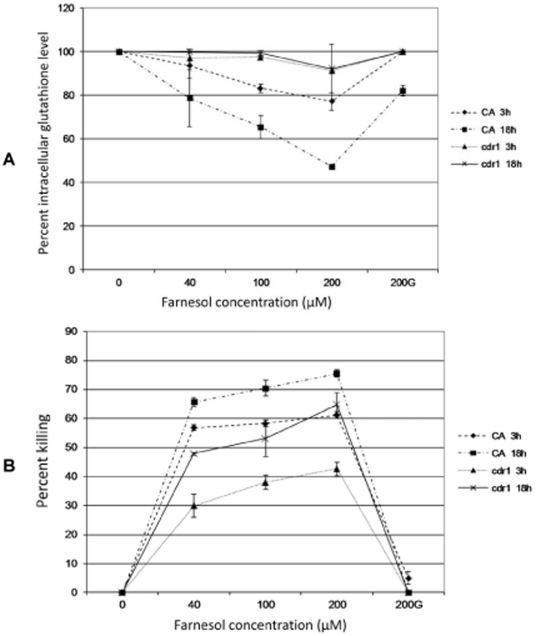
Effect of farnesol on intracellular glutathione levels and viability in CA and *cdr1* in the presence and absence of exogenous glutathione following 3 h and 18 h exposure to increasing farnesol concentrations. (**A**) Significant decrease in glutathione levels in CA proportional to farnesol concentration and time of exposure with minimal decrease in glutathione in *cdr1*. Exogenous glutathione (G) inhibited drop in intracellular levels in both strains. Error bars indicate the standard errors of the means. (**B**) Farnesol concentration and exposure time-dependent killing of *C. albicans* with *cdr1* exhibiting decreased susceptibility. However, no killing was observed upon supplementation with exogenous glutathione (G). Error bars indicate the standard errors of the means.

As conjugation of farnesol with reduced GSH is expected to deplete its levels, the total cellular glutathione concentration was comparatively measured in control (100%) and farnesol-exposed cells. Results demonstrated that farnesol exposure resulted in significant decrease (p<0.05) in total cellular glutathione content proportional to farnesol concentration and time of exposure with maximum drop of >50% noted at 18 h exposure to 200 µM farnesol. However, the drop in glutathione levels in CA was inhibited upon supplementation of exogenous GSH at 3 h, whereas at 18 h exposure, the drop was only 18% compared to the initial 50% in the absence of glutathione (Fig. 2A). In contrast, *cdr1* demonstrated sustained levels of intracellular glutathione in the presence of farnesol with a slight drop at 200 µM farnesol which was restored to normal in the presence of glutathione.

### MTS viability assay

The MTS metabolic assay was performed to assess farnesol's killing effect on *C. albicans*. Cell proliferation following exposure to increasing concentrations of farnesol (10, 40, 100 and 200 µM) was assessed by measuring absorbance following color development. Results indicated a significant (p<0.05) killing effect for farnesol on CA proportional to farnesol concentration and exposure time with maximum killing at 18 h exposure to 200 µM. Although the trend was similar, the killing of *cdr1* was significantly less (p<0.05) drastic. However, no or minimal killing was observed with either strain upon glutathione supplementation (Fig. 2B). To confirm the involvement of *CDR1* in farnesol-dependent killing, viability assays were also performed on the *CDR1*-complemented strain which exhibited approximately 52% killing at 100F following 3 h exposure, a profile similar to that for the wild type strain (∼58% killing) and control strain (∼59% killing) compared to *cdr1* (∼38% killing). The similarity in farnesol-dependent killing between the revertant and wild type strains was also consistent under all conditions tested (different farnesol concentrations and exposure times) confirming successful complementation of *CDR1*.

Assays using various *C. albicans* cell density as well as time course killing experiments were also performed. Results from these experiments demonstrated that *C. albicans* killing was inversely proportional to its cell density (Fig. S2) and proportional to time of exposure to farnesol (Fig. S3).

### Viability assay by plate dilution

In addition to the MTS metabolic assay *C. albicans* viability was also assessed using plate dilution and colony forming units (CFU) counts. The results based on percent killing from these experiments demonstrated a similar farnesol-dependent killing effect on *C. albicans* (Fig. S4). Viability assays using plating were also performed with glutathione supplementation. CFU counts confirmed the ability of exogenous glutathione to reduce the farnesol-induced killing at all farnesol concentrations and exposure times tested (Fig. S4).

### Overexpression of *CDR1*

**Figure 3:**
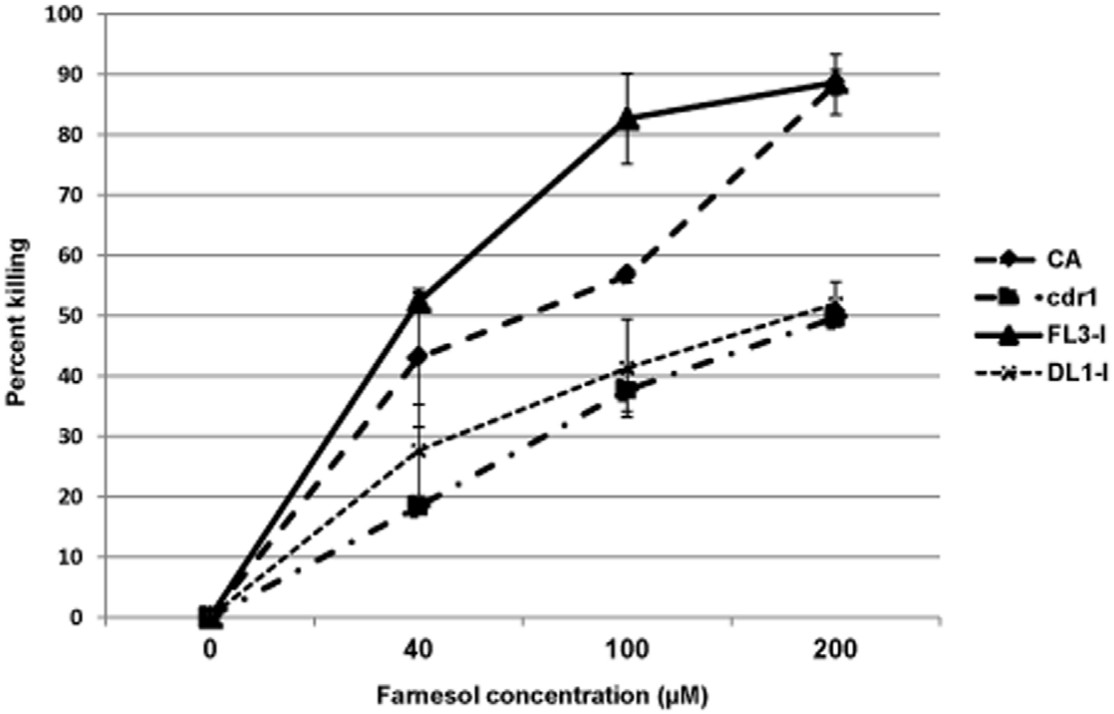
Effect of *CDR1* overexpression on the response to farnesol toxicity using the MTS viability assay. Significant increase in susceptibility to farnesol in FL3 strain following induction of *CDR1* overexpression with the strains lacking the *CDR1* gene demonstrating enhanced tolerance. Error bars indicate the standard errors of the means. p<0.05.

To confirm the involvement of *CDR1* in farnesol cytotoxicity, the effect of farnesol on a strain overexpressing *CDR1* was tested. *C. albicans* FL3 and DL1 cells were grown in medium containing GlcNAc as carbon source to induce the *HEX1* promoter and the expression of *CDR1*. Results from the MTS assay demonstrated that at 100F the *CDR1* overexpressing strain exhibited approximately 30% enhanced susceptibility to farnesol compared to wild-type and over 50% increase compared to its control strain DL1 and *cdr1* (Fig. 3).

The overexpression of *CDR1* in the FL3 strain was confirmed by RT-PCR analysis where gene expression analysis demonstrated significant increase in *CDR1* expression in the induced FL3 strain compared to the wild-type whereas, as expected, no expression was seen in the *cdr1* and DL1 strains (Fig. S5).

### GSH/GSSG homeostatis and redox balance

**Figure 4:**
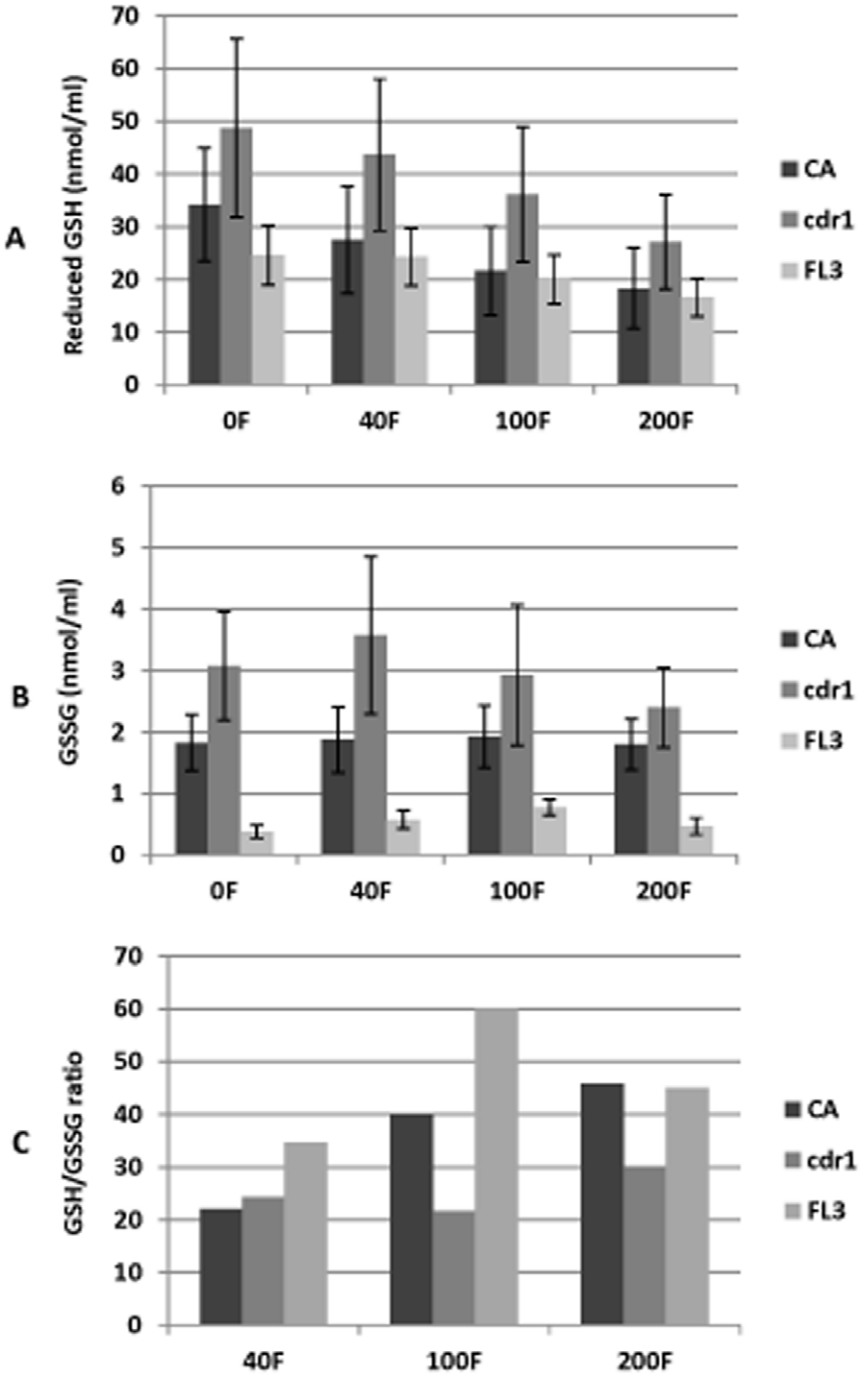
Effect of farnesol on intracellular GSH/GSSG homeostatis and redox balance. Significant reduction in GSH levels proportional to farnesol concentration with FL3 exhibiting lowest levels and highest disruption in ratio whereas *cdr1* had highest GSH levels with minimal changes in ratio noted. Error bars indicate the standard errors of the means.

No significant changes in reduced GSG and GSSG content were noted when cells where exposed to farnesol for 3 h. However, significant changes were noted following 18 h exposure in PBS. As can be seen in Fig. 4, for all strains tested, the reduced GSH levels decreased proportional to farnesol concentration (Fig. 4A). Strikingly, at 0F the FL3 strain exhibited the lowest levels of GSH whereas *cdr1* the maximum levels. This profile was consistent in the presence of all farnesol concentrations tested. Similarly, the intracellular GSSG content at 0F and all farnesol concentrations was the highest in *cdr1* and significantly lower in FL3 (Fig. 4B). Based on the averaged content of reduced GSH and GSSG, the maximum disruption in ratio was seen with FL3 followed by the CA whereas no or minimal changes were noted in *cdr1* (Fig. 4C).

### ROS accumulation

**Figure 5:**
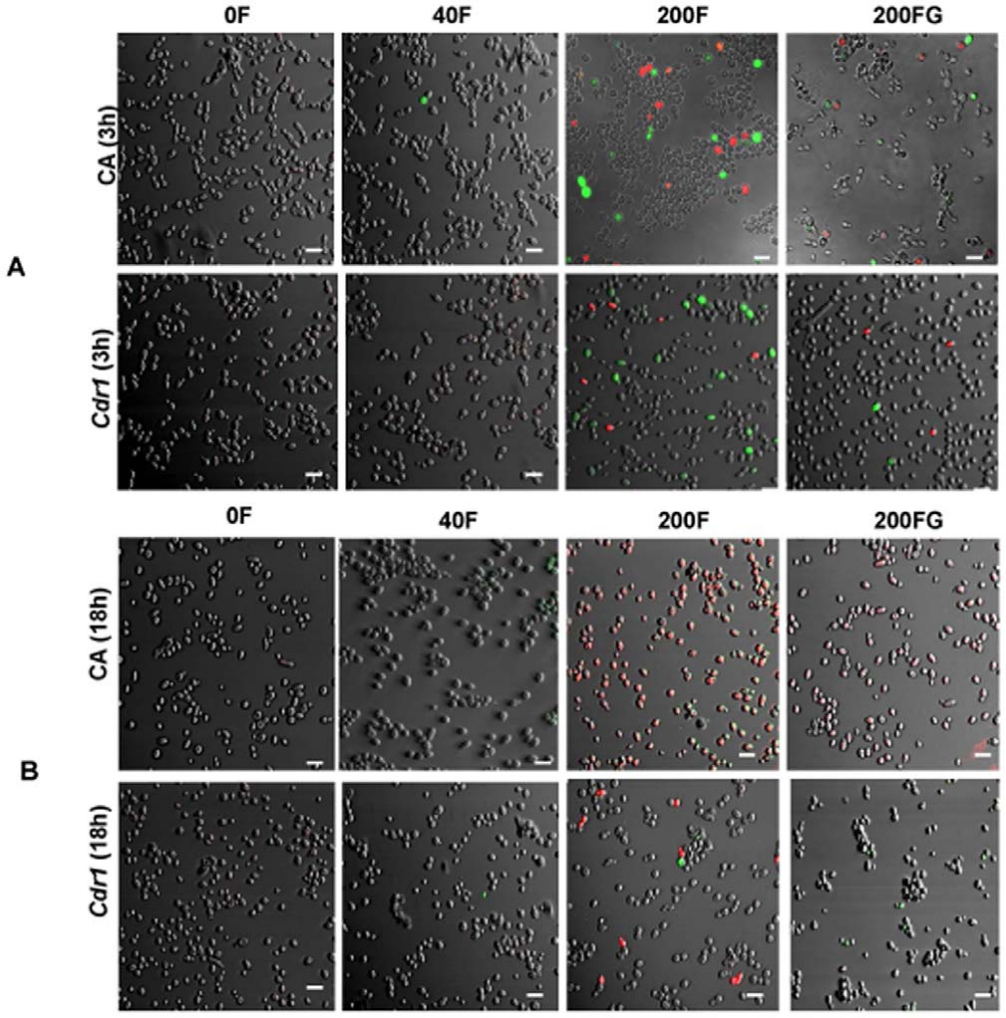
Representative confocal scanning laser fluorescent images of CA and *cdr1* cells stained for ROS accumulation and necrosis following exposure to 40 and 200 µM farnesol in the absence (F) and presence (FG) of exogenous glutathione. (**A**) Minimal green (ROS accumulation) and red (dead) fluorescence is seen in CA and *cdr1* following 3 h exposure to farnesol whereas (**B**) the majority of CA cells were red following 18 h exposure with significant decrease in fluorescence seen upon exogenous glutathione supplementation). In contrast, minimal fluorescence was seen in *cdr1* cells at 18 h. Bar represents 20 µm. p<0.05.

The effect of farnesol was also evaluated microscopically *via* assessment of ROS accumulation and necrosis following 3 h and 18 h exposure of CA and *cdr1* cells in the absence and presence of glutathione. Following incubation, cells were stained using a combination of DCDHF-DA and propidium iodide. Confocal images demonstrated no or minimal green or red fluorescence in both strains following 3 h exposure to 40 µM farnesol (Fig. 5A). However, at 200 µM a comparable number of CA and *cdr1* cells exhibited green or red fluorescence which was reduced upon glutathione supplementation (Fig. 5A). Following 18 h exposure, minimal (<1%) green fluorescence was seen in *cdr1* at 40 µM with more CA (5%) cells showing green fluorescence at that concentration (Fig. 5B). In contrast, at 200 µM, the majority (>90%) of CA cells were red with few green cells compared to only few green (10%) and red (<2%) *cdr1* cells (Fig. 5B). However, in the presence of glutathione, significantly less fluorescent CA (50% red) and *cdr1* (<1% green) cells were seen (Fig. 5B). Values represent the average counts of cells from 10 fields.

### Caspases activation

**Figure 6:**
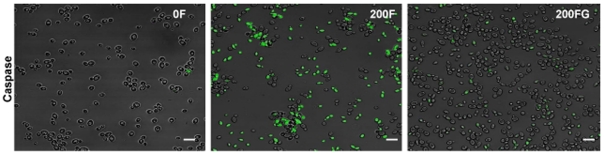
Representative confocal scanning laser fluorescent images of *C. albicans* cells stained for intracellular caspase activation following treatment with 200 µM farnesol in the absence (F) and presence (FG) of exogenous glutathione. Significant activation of intracellular caspases in farnesol-exposed cells as indicated by green fluorescence with minimal fluorescence in cells treated with glutathione. Bar represents 20 µm.

As the presence of activated intracellular caspases is indicative of activation of an apoptotic process, CA cells were exposed to 200 µM farnesol in the absence and presence of exogenous glutathione to assess whether glutathione modulates the apoptotic effect of farnesol on the cell. Images revealed the presence of green fluorescence in the farnesol-exposed cells indicating activation of intracellular caspases. However, glutathione addition significantly inhibited the activation of intracellular caspases (Fig. 6).

### Changes in glutathione metabolic enzymes activity

**Figure 7:**
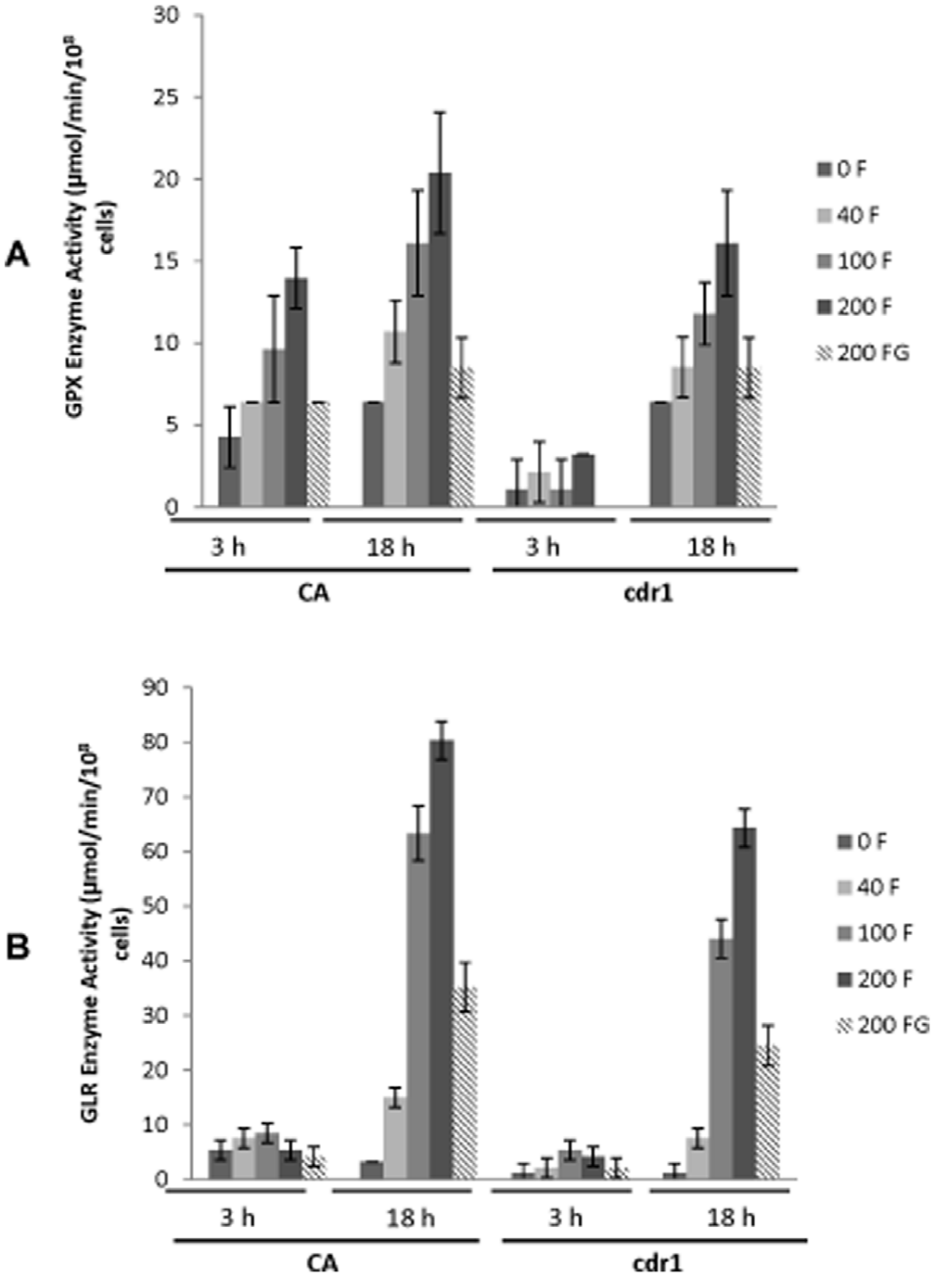
Effect of farnesol on glutathione peroxidase (GPX) and glutathione reductase (GLR) intracellular enzyme activity in CA and *cdr1* following 3 h and 18 h exposure to increasing concentrations of farnesol. (**A**) Farnesol dose-dependent increase in GPX activity in CA following 3 h and 18 h exposure to farnesol. Similar increase in *cdr1* at 18 h with no change at 3 h (**B**) Increase in GLR activity in CA and *cdr1* at 18 h with *cdr1* exhibiting less activity than CA. In contrast, no effect on GLR activity was noted in both strains following 3 h exposure to farnesol. However, activity of both enzymes was restored to near baseline levels in the presence of exogenous glutathione. In all experiments, GSH supplementation maintained enzyme activity at normal baseline levels. Error bars indicate the standard errors of the means. p<0.05.

Depletion of intracellular GSH levels due to farnesol exposure is expected to activate enzymatic reactions in the glutamyl cycle as a compensatory mechanism for depletion. Therefore, the levels of activity of glutathione peroxidase (GPX) and glutathione reductase (GLR), key enzymes involved in glutathione metabolism were measured. Results from these assays demonstrated an increase in intracellular GPX activity in CA proportional to farnesol concentration and exposure time (Fig. 7A). In contrast, no change in activity was noted with *cdr1* following 3 h exposure to farnesol with increase observed at 18 h. Upon glutathione supplementation however, the level of GPX enzyme in both strains was reduced to near baseline levels at both time points. Similar to GPX, in both strains, GLR activity significantly increased in a manner proportional to farnesol concentration following 18 h exposure, however, at 3 h the level of GLR activity remained at baseline levels (Fig. 7B). In general and consistent with other assays, the increase in GLR activity was higher in CA compared to *cdr1* with levels returning to baseline with glutathione at 3 h exposure. In contrast, at 18 h, although GLR level was drastically reduced with glutathione, it remained significantly higher than baseline (Fig. 7B).

The observed differential increase in both enzyme activities in response to farnesol was consistent with results from RT-PCR analysis of *C. albicans GPX2* and *GLR1* genes following 3 and 18 h exposure to farnesol (Fig. S6).

### Expression of the *SOD* genes

**Figure 8:**
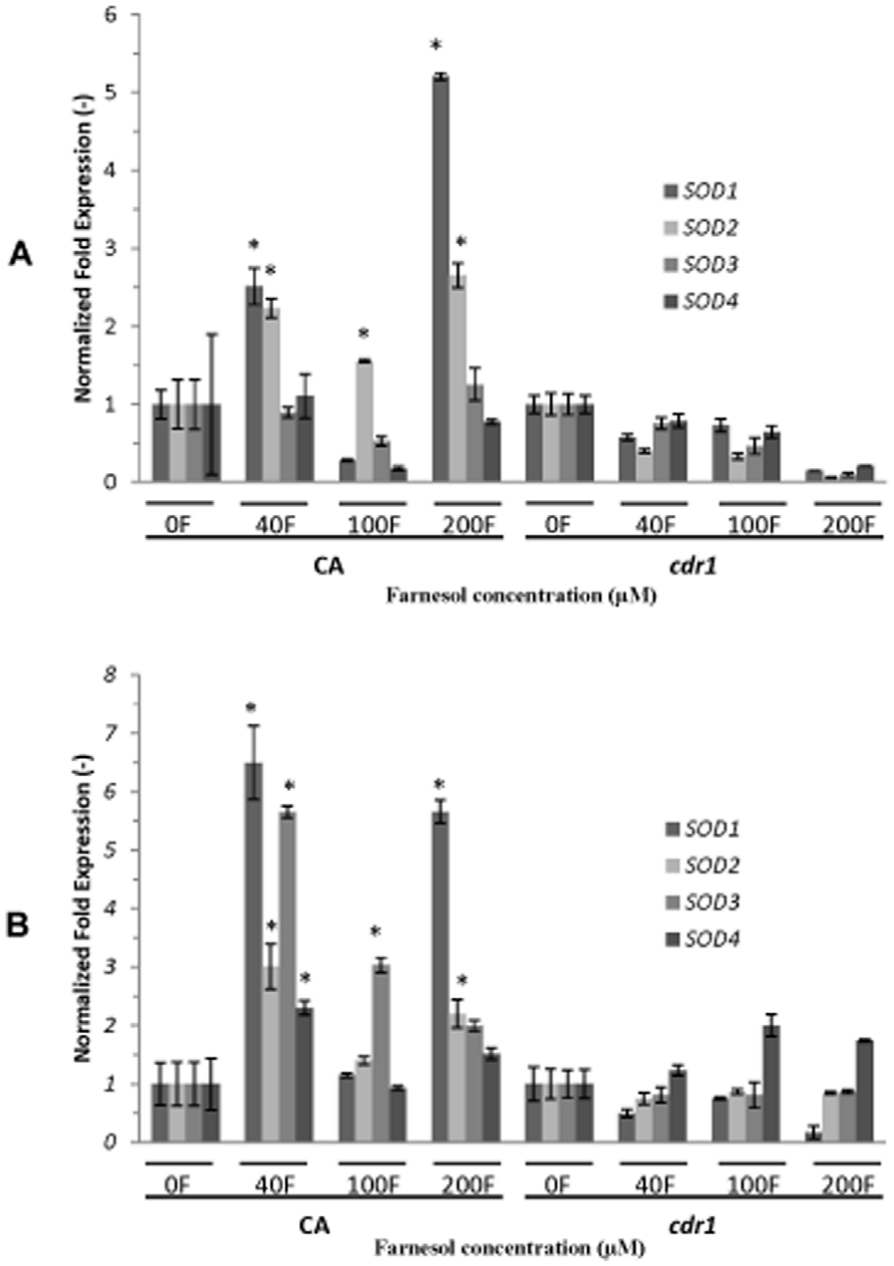
Effect of farnesol on the expression of the *SOD1-4* genes in CA and *cdr1* cells following 3 h and 18 h exposure to increasing concentrations of farnesol as determined by qRT-PCR analysis. (**A**) Significant differential expression in *SOD* genes in CA at 3 h with no significant variation in *cdr1*. (**B**) Similar but more pronounced expression profiles were seen at 18 h in both strains. Expression is normalized against *ACT1* as reference gene. Data represent the average of 3 independent experiments performed in triplicate. Error bars indicate the standard errors of the means. p<0.05.

As the *C. albicans* Sods are the primary enzymatic antioxidants involved in protection against ROS, it is expected that their expression is modulated by ROS accumulation in the cell. Therefore, to evaluate the response of *C. albicans* to oxidative stress, expression of the *SOD* genes was assessed in CA and *cdr1* following 3 (Fig. 8A) and 18 h (Fig. 8B) exposure to farnesol. Results from these experiments demonstrated variable but significant upregulation in the *SOD* genes in the parent strain at both exposure time points with or without GSH supplementation (data not shown). In contrast, no significant changes in expression were seen in *cdr1* following 3 h or 18 h exposure (Fig. 8A and B).

## Discussion

Several key physiological roles have been described for the quorum-sensing molecule farnesol in *C. albicans* cell cycle. However, our previous investigations have shown that above threshold concentrations, farnesol triggers a classical apoptotic process causing death to the fungal cell [11]. Since farnesol is described to be increasingly secreted by the fungus in aging cultures, it is conceivable to speculate that through the regulation of farnesol production, *C. albicans* cells may have developed a mechanism of programmed cell death with evolutionary advantages [35]. Therefore, the characterization of the mechanistic switches that regulate active death processes in *C. albicans*, may lead to the development of novel antifungal agents that switch on endogenous cell suicide.

This current study was undertaken in order to elucidate a defined mechanism behind the demonstrated farnesol-induced apoptotic process in *C. albicans*. Specifically, experiments were designed to validate the hypothesis that the mechanism of farnesol toxicity involves consumption of the intracellular antioxidant GSH and modulation of Cdr1p activity resulting in depletion of total glutathione, oxidative stress and ultimately fungal cell death. The effect of farnesol on intracellular glutathione level was clearly demonstrated by measuring the levels of total glutathione with simultaneous measurements of glutathione-metabolizing activities in response to farnesol treatment. The combined results from these experiments revealed a farnesol concentration and exposure time-dependent decrease in total glutathione levels, concomitant with decrease in *C. albicans* viability (Fig. 2). However, exogenous GSH supplementation not only maintained the intracellular glutathione level and enhanced survival (Fig. 2), but also significantly decreased ROS accumulation and caspase activation (Fig. 5 and 6). It is interesting to note that although GSH supplementation almost completely inhibited killing by farnesol based on results from the MTS assay (Fig. 2B), these results were not consistent with those from the viability assay used based on plating and CFU counts. Although CFU counts clearly demonstrated enhanced tolerance to farnesol with GSH at all conditions tested (Fig. S4), the effect was not as drastic as that seen with MTS (Fig. 2B). However, it is important to note that cells demonstrated enhanced metabolic activity in the presence of GSH in the absence of farnesol based on noted increase in absorbances (data not shown). Therefore, the significant effect seen for GSH with MTS assay is likely due to the enhanced metabolic activity of surviving cells and therefore, assessment of viability based on CFU counts would be the more accurate measure of GSH effect on cell survival.

To associate the observed decrease in GSH levels with Cdr1p activity, gene expression studies were performed where *CDR1* expression was shown to be significantly upregulated in the cells exposed to farnesol in a concentration-dependent manner (Fig. 1). Although the observed increase in *CDR1* expression did not change with glutathione supplementation, these results are expected as *CDR1* expression is induced by formation of glutathione conjugates upon farnesol exposure. Therefore, although exogenous GSH prevents intracellular depletion, the impact of farnesol exposure on *CDR1* expression remains. These findings demonstrating farnesol modulation of expression of ABC transporters were recently confirmed by a study by Sharama *et al.* [36].

The involvement of Cdr1p was further established using a *C. albicans* null mutant strain (*cdr1*) lacking *CDR1*. The combined results demonstrated that, in contrast to CA, no significant decline in intracellular glutathione levels was seen in *cdr1*, concomitant with decreased farnesol-dependent killing at all concentrations and time points tested. Alternatively, overexpression of *CDR1* resulted in enhanced killing of farnesol (30% increase) in FL3 cells following induction of *CDR1* (Fig. 3). These findings are intriguing as paradoxically, the *cdr1* strain is typically hypersusceptible to azole drugs and several metabolic inhibitors and overexpression of *CDR1* has been shown to result in increased resistance to fluconazole [27], [37], [38].

Collectively, these findings provide direct evidence for the involvement of Cdr1p activity in farnesol cytotoxicity. It is important to note however, that although overexpression of *CDR1* would be expected to confer resistance to fluconazole, in our experience fluconazole MIC did not dramatically increase upon *CDR1* induction. However, although the MIC remained well within the susceptible range, it was consistently higher following induction. These results were not surprising as similarly, using the microbroth dilution method Niimi *et al*. [30] also did not find significant increase in fluconazole MIC. Furthermore, in our study, the observed increase in farnesol-dependent killing of the induced strain to farnesol was approximately 30%. Combined, these findings indicate that although the level of *CDR1* expression obtained from the induction process was sufficient to confirm a key role for *CDR1* in farnesol cytotoxicity, it was not high enough to confer resistance to fluconazole.

In contrast to *cdr1*, the *cdr2* mutant demonstrated minimal decrease in susceptibility to farnesol. The differences in farnesol susceptibility between these mutant strains are not surprising, as despite a high level of structural conservation, Cdr1p and Cdr2p were shown to exhibit major functional differences and susceptibility profiles to various antifungal agents, suggesting distinct biological functions [27], [39]. Specifically, Cdr1p was shown to mediate the transport of human steroid hormone [37]. In addition, it is important to note that in the absence of *CDR2*, *CDR1* remains functional and therefore a *cdr2* mutant would be expected to behave similarly to the wild type. Combined these findings indicate that GSH efflux from cells is positively correlated with the level of *CDR1* expression.

**Figure 9:**
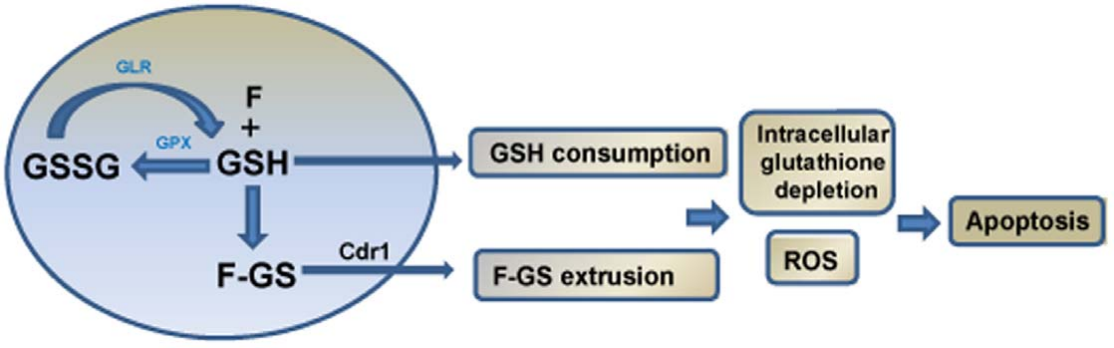
Proposed mechanism of farnesol cytotoxicity in *C. albicans*. Farnesol conjugates with reduced glutathione (GSH) forming glutathione S-conjugate (F-GS) concomitant with oxidation of GSH to the disulfide form (GSSG), a reaction catalyzed by GPX. GSSG is reduced to GSH by GLR as a recycling mechanism to maintain GSH levels. However, the oxidation and consumption of intracellular GSH coupled with Cdr1p-mediated export of F-GS results in depletion of intracellular GSH, disruption of the redox balance and oxidative stress leading to apoptosis.

In the cell, glutathione is synthesized and metabolized by the reactions of the γ-glutamyl cycle [18]. The two key enzymes catalyzing these reactions are glutathione peroxidase (GPX) which catalyzes the reduction of hydroperoxides by reduced glutathione and glutathione reductase (GLR), a flavoprotein that catalyzes the NADPH-dependent reduction of oxidized glutathione (GSSG) to glutathione (GSH) [18]. GPX responds to oxidative stress increasing cellular capability to produce GSSG, which would need to be reduced back to GSH by the GLR enzyme or extruded out of the cell by the action of efflux pumps (Fig. 9). In combination, these two cellular strategies maintain adequate levels of reduced cellular GSH. Therefore, increased GLR activity in the cell serves as a compensatory mechanism by recycling GSSG to GSH in order to maintain a high GSH/GSSG ratio. This ability of both GPX and GLR to transcriptionally respond to oxidative stress suggests the presence of a coordinated antioxidant cellular response.

Therefore, in this study, experiments were also performed to monitor the activity of GPX and GLR following initial and prolonged exposure to farnesol. Results from these experiments demonstrated a conspicuous activation of GPX in the wild-type following 18 h exposure proportional to farnesol concentration, with a less drastic increase following 3 h (Fig. 7A). In contrast, although a similar increase in GPX activity was seen for the *cdr1* mutant at 18 h, no change was noted upon initial exposure (3 h) indicating no change in intracellular GSH levels at that time point, consistent with the results from GSH measurement (Fig. 7A). Similarly, GLR activity was increased in both the parent and mutant strain at 18 h with no increase in activity detected upon 3 h exposure (Fig. 7B). These exposure time-associated differences in GPX and GLR activity are expected as GPX is the first enzyme to be activated in the cycle in response to farnesol to conjugate toxic compounds to GSG producing GSSG. However, as GSSG levels increase and GSH is depleted over time, GLR is activated as a compensatory recycling system to maintain levels of reduced GSH. Farnesol-induced depletion of intracellular glutathione levels was corroborated by the findings demonstrating baseline levels of both GPX and GLR upon exogenous glutathione supplementation (Fig. 7).

In all eukaryotic cells, the cytoplasmic GSH content as well as the GSH/GSSG redox status must be strictly regulated, since a drastic reduction of GSH and the concomitant imbalance in GSH/GSSG ratio causes severe cell mortality. This disruption in cellular redox homeostatis was demonstrated using biochemical assays to assess the disruption in GSH/GSSG ratio. Using biochemical assays, results demonstrated significant reduction in reduced GSH content proportional to farnesol concentration with the strain over-expressing *CDR1* demonstrating lowest intracellular levels and the strain lacking *CDR1* demonstrating highest levels (Fig. 4). Based on the content of reduced GSH and GSSG, the maximum disruption in ratio was seen with FL3 followed by the CA whereas no or minimal changes were noted in the *cdr1* (Fig. 4C). Taken together, these findings support the involvement of farnesol-induced decrease in reduced GSH levels leading to disruption of cellular redox status.

The increased resistance of *cdr1* to oxidative stress conferred upon farnesol exposure was corroborated by microscopic images demonstrating decrease presence of intracellular ROS accumulation and apoptosis. Although the presence of cells under oxidative stress and apoptosis was comparable for CA and *cdr1* at 3 h exposure, at 18 h a dramatic difference in response to 200 µM farnesol was noted between the two strains where the majority of CA cells were dead (Fig. 5). The counter effect of exogenous glutathione however, was seen in both strains under all conditions tested (Fig. 5). Interestingly, although at 40 µM farnesol resulted in significant killing of *C. albicans* as demonstrated by viability assays, the microscopic images are not reflective of that level of adverse effect at that concentration. However, it is important to note that killing assays were performed in PBS in 96-well microtiter plates whereas for ROS staining, the cells were grown in media in the presence of farnesol. Nevertheless, the trend of the results from the various assays is consistent with the notion that farnesol exerts a concentration and exposure time-dependent cytotoxic effect which is alleviated by exogenous glutathione and is less dramatic in the absence of Cdr1p.

As clearly demonstrated by us and others, farnesol stimulates the production of ROS which can damage almost every essential cellular component [11], [14], [40], [41]. Therefore, fungal cells have evolved specific defense mechanisms to neutralize ROS. The primary enzymatic antioxidants involved in protection against ROS are a family of cytoplasmic and mitochondrial superoxide dismutases (Cu-Zn and Mn-SODs), which act directly as ROS detoxifiers [14], [41]–[43]. The combined indicators of a decreased response to oxidative stress in the *cdr1* strain compared to the parent strain prompted us to evaluate the gene expression profile of enzymes crucial for defense against oxidative stress in *C. albicans*. Quantitative RT-PCR analysis demonstrated significant up-regulation of the *SOD1-4* genes in the parent strains in response to 3 and 18 h exposure to farnesol (Fig. 8). Strikingly, no significant differences in the expression of the *SOD* genes was noted in the *cdr1* strain, supporting the overall indicators of decreased response to oxidative stress in the absence of Cdr1p.

The mechanism behind the noted differences in the expression of the various *SOD* genes is not quite clear. However, Sod2p is primarily responsible for scavenging intracellularly produced superoxides and therefore, *SOD2* would be expected to be upregulated in response to intracellular ROS accumulation the result of farnesol exposure [43]. In addition, *C. albicans* seems to coordinate the production of CuZnSOD (*SOD1*) and MnSOD (*SOD2*) in a reciprocal manner which is seen in our findings [43]. On the other hand, *SOD3* was shown not to be stimulated by drug-induced oxidative stress but rather to be involved in the protection against reactive oxygen species during the stationary phase and therefore, is induced upon entry and during stationary phase [43], [44]. This is also consistent with our results where *SOD3* expression seemed to increase at the 18 h growth period but not at 3 h. Therefore, the observed overexpression of this gene may not be in response to farnesol but rather to entry into a stationary phase. Alternatively, although the function of the Sod4p isoenzyme remains to be established, *SOD4* is regulated during phenotypic switching [43]. Therefore, *SOD4* expression is not expected to be affected as the experiments were performed on yeast cultures. The ambiguity of the expression profiles of the *SOD* genes in response to farnesol is further complicated by the observed response upon exogenous glutathione supplementation, where no significant changes were noted in the absence and presence of glutathione. Although a decrease in expression would be expected, it is important to note that GSH supplementation alleviated the oxidative stress exerted by farnesol on the cell as shown by the results from the various experiments but did not completely neutralize the damaging effect. Therefore, it is more logical to expect that the cell will continue to respond to the stress *via SOD* gene regulation.

Taken together, the findings presented here indicate that the enhanced overall resistance of the *cdr1* mutant to farnesol-dependent killing is prominent during early stages of exposure to moderate farnesol concentrations. As the concentration of farnesol increases and exposure time is prolonged, farnesol toxicity increases in the *cdr1* strain. However, it is important to note that the hydrophobic nature of farnesol favors its accumulation in microbial cell membranes, resulting in disruption of membrane integrity, as we have previously demonstrated in *C. albicans* and in the bacterial pathogen *Staphylococcus aureus* [11], [45]. Therefore, it is likely that at high concentration, farnesol causes extensive disruption of the cell membrane leading to immediate cell death or necrosis. Alternatively, at lower concentrations, farnesol toxicity is mediated *via* disruption of the intracellular redox balance and ROS accumulation. Therefore, it is probable that necrosis and apoptosis are occurring simultaneously, which may explain the initial enhanced toxicity of farnesol observed in the *cdr1* mutant.

In conclusion, the combined findings from this study provide evidence that farnesol reduces intracellular levels of GSH *via* Cdr1p export from the intracellular compartment, resulting in glutathione depletion and disruption of the redox balance. However, absence of *CDR1* allows cells to maintain appropriate levels of total intracellular GSH and redox potential promoting cell survival and function. Although Cdr1p may serve as an immediate mechanism for the cell to eliminate oxidized species, this process, if stimulated for a prolonged period, may reduce the total intracellular thiol pool and paradoxically have an undesirable effect on the intracellular ratio of GSH/GSSG and cell function. We would like to note that the instability of the farnesol-GSH conjugate formed has made it problematic to measure its levels intracellularly and extracellularly and therefore, this aspect remains speculative. However, we expect the novel findings presented here to pave the way for more studies where sensitive methods can be applied to confirm the formation of farnesol-GSH conjugates.

To our knowledge, this is the first study elucidating a defined mechanism behind farnesol-induced apoptosis in *C. albicans*. These findings will enable a better understanding of the molecular mechanisms underlying farnesol-mediated cytotoxicity in eukaryotic cells. Therefore, further investigations are warranted to explore the potential of this redox-cycling agent as an alternative antifungal agent.

## Supporting Information

Figure S1 Complementation of *CDR1* inactivation analyzed by (**A**) Western analysis with a Cdr1p antibody. Wild type Cdr1p signal is restored in the revertant DSY4481 and (**B**) drug resistance phenotype. Wild type fluphenazine susceptibility is restored in the revertants (two independent transformants were spotted). DSY4481. Strains designation: DSY4310: CAF4-2 transformed with CIP10; DSY4480: DSY449 transformed with CIP10; DSY4481: DSY449 transformed with pDS1765.
(TIF)

Figure S2 *C. albicans* cell density killing curve. Percent killing *of C. albicans* by farnesol was inversely proportional to cell density. Error bars indicate the standard errors of the means.
(TIF)

Figure S3 Time course killing of *C. albicans* by farnesol. Percent killing of *C. albicans* was proportional to time of exposure to farnesol. However, the difference in level of killing between the different time points was not significant. Error bars indicate the standard errors of the means.
(TIF)

Figure S4 *C. albicans* viability as assessed by plate dilution method. Percent killing of *C. albicans* based on CFU counts is proportional to farnesol concentration and time of exposure with enhanced tolerance to farnesol upon GSH supplementation. Error bars indicate the standard errors of the means.
(TIF)

Figure S5 RT-PCR gene expression analysis of *C. albicans* strains following induction of *CDR1*. Significant increase in the expression of *CDR1* in the induced FL3 compared to wild-type with no expression detected in the strains lacking the *CDR1* gene (*cdr1* and DL1).
(TIF)

Figure S6 RT-PCR gene expression analysis of *C. albicans GPX2* and *GLR1* following 3 and 18 h exposure to farnesol. Significant increase in the expression of both genes at 18 h whereas only *GPX2* is increased at 3 h.
(TIF)
