# Supplementary figures and images for: Farnesol-Induced Apoptosis in Candida albicans Is Mediated by Cdr1-p Extrusion and Depletion of Intracellular Glutathione
Source: PLoS One. 2011 Dec 19;6(12):e28830. doi: 10.1371/journal.pone.0028830 (PMC3242750; doi:10.1371/journal.pone.0028830)

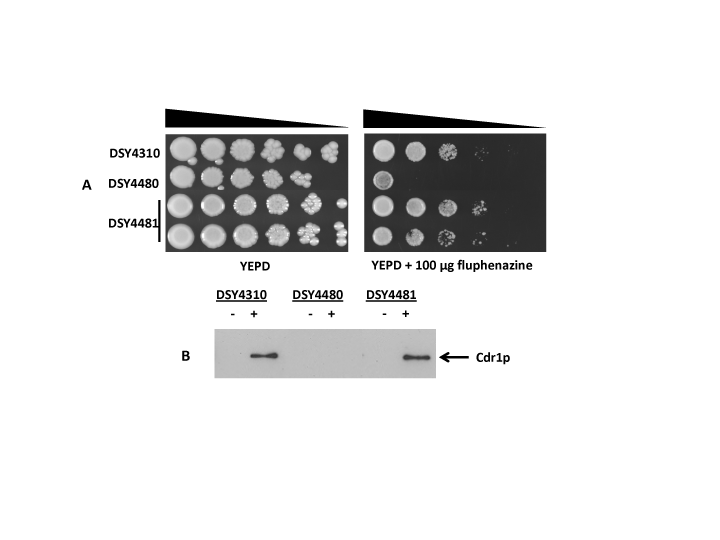

Supplement: Figure S1 — Complementation of CDR1 inactivation analyzed by (A) Western analysis with a Cdr1p antibody. Wild type Cdr1p signal is restored in the revertant DSY4481 and (B) drug resistance phenotype. Wild type fluphenazine susceptibility is restored in the revertants (two independent transformants were spotted). DSY4481. Strains designation: DSY4310: CAF4-2 transformed with CIP10; DSY4480: DSY449 transformed with CIP10; DSY4481: DSY449 transformed with pDS1765. (TIF) [file pone.0028830.s001.tif]

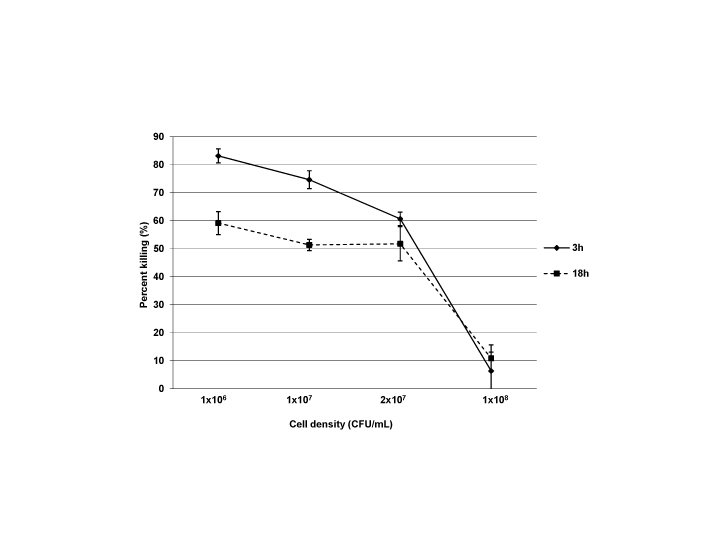

Supplement: Figure S2 — C. albicans cell density killing curve. Percent killing of C. albicans by farnesol was inversely proportional to cell density. Error bars indicate the standard errors of the means. (TIF) [file pone.0028830.s002.tif]

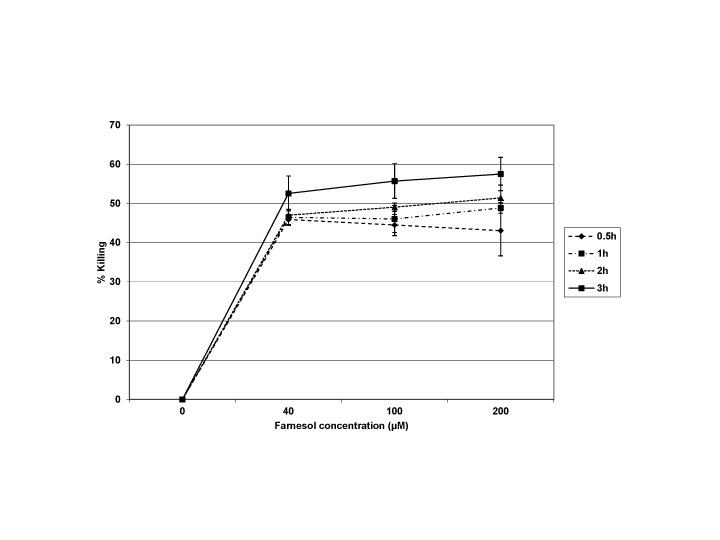

Supplement: Figure S3 — Time course killing of C. albicans by farnesol. Percent killing of C. albicans was proportional to time of exposure to farnesol. However, the difference in level of killing between the different time points was not significant. Error bars indicate the standard errors of the means. (TIF) [file pone.0028830.s003.tif]

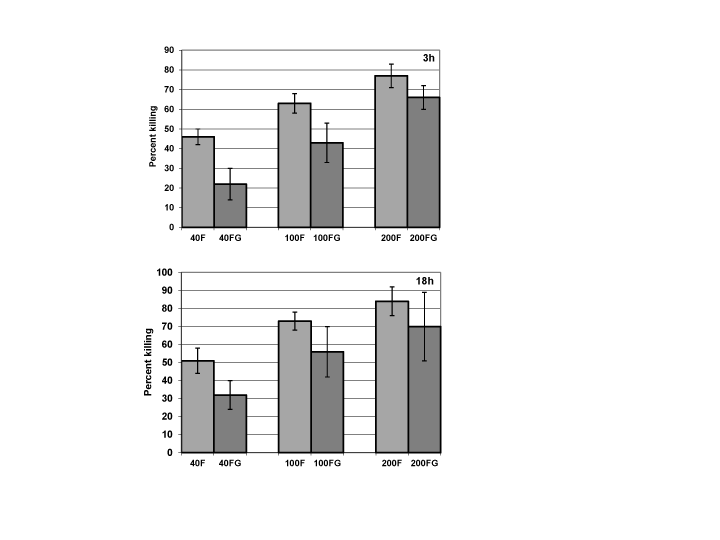

Supplement: Figure S4 — C. albicans viability as assessed by plate dilution method. Percent killing of C. albicans based on CFU counts is proportional to farnesol concentration and time of exposure with enhanced tolerance to farnesol upon GSH supplementation. Error bars indicate the standard errors of the means. (TIF) [file pone.0028830.s004.tif]

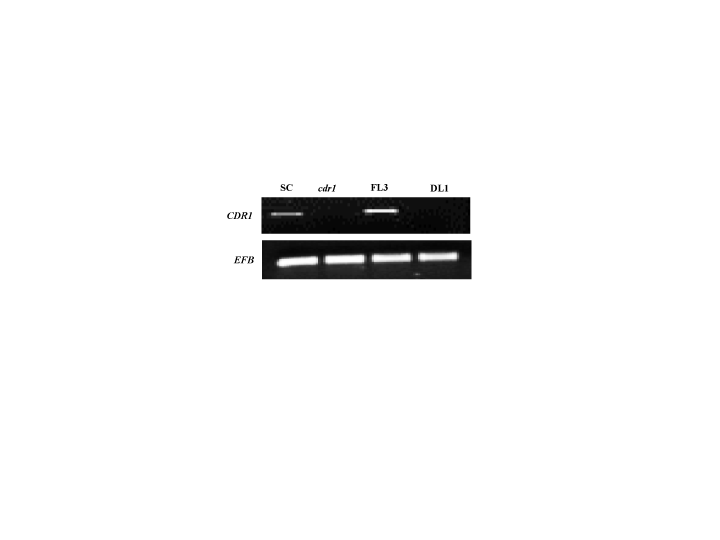

Supplement: Figure S5 — RT-PCR gene expression analysis of C. albicans strains following induction of CDR1. Significant increase in the expression of CDR1 in the induced FL3 compared to wild-type with no expression detected in the strains lacking the CDR1 gene (cdr1 and DL1). (TIF) [file pone.0028830.s005.tif]

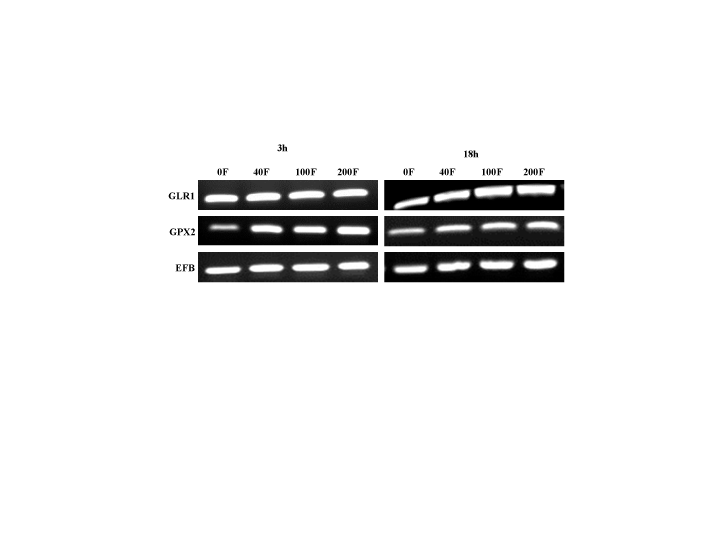

Supplement: Figure S6 — RT-PCR gene expression analysis of C. albicans GPX2 and GLR1 following 3 and 18 h exposure to farnesol. Significant increase in the expression of both genes at 18 h whereas only GPX2 is increased at 3 h. (TIF) [file pone.0028830.s006.tif]
